# Inflammatory response-based prognostication and personalized therapy decisions in clear cell renal cell cancer to aid precision oncology

**DOI:** 10.1186/s12920-023-01687-5

**Published:** 2023-10-26

**Authors:** Weimin Zhong, Huijing Chen, Jiayi Yang, Chaoqun Huang, Yao Lin, Jiyi Huang

**Affiliations:** 1 Central laboratory, The Fifth Hospital of Xiamen, Xiamen, 361101 Fujian Province China; 2 Central Laboratory at The Second Affiliated Hospital of Fujian Traditional Chinese Medical University, Collaborative Innovation Center for Rehabilitation Technology, Fujian University of Traditional Chinese Medicine, Fuzhou, 350122 China; 3 Department of Nephrology, The Fifth Hospital of Xiamen, Xiamen, 361101 Fujian Province People’s Republic of China

**Keywords:** Inflammatory response, Clear cell renal cell cancer, Categorization, Prognosis, Targeted drugs, Immuno-oncology, Pan-cancer

## Abstract

**Objective:**

The impact of inflammatory response on tumor development and therapeutic response is of significant importance in clear cell renal cell carcinoma (ccRCC). The customization of specialized prognostication approaches and the exploration of supplementary treatment options hold critical clinical implications in relation to the inflammatory response.

**Methods:**

In the present study, unsupervised clustering was implemented on TCGA-KIRC tumors using transcriptome profiles of inflammatory response genes, which was then validated in two ccRCC datasets (E-MATB-1980 and ICGC) and two immunotherapy datasets (IMvigor210 and Liu et al.) via SubMap and NTP algorithms. Combining co-expression and LASSO analyses, inflammatory response-based scoring system was defined, which was evaluated in pan-cancer.

**Results:**

Three reproducible inflammatory response subtypes (named IR1, IR2 and IR3) were determined and independently verified, each exhibiting distinct molecular, clinical, and immunological characteristics. Among these subtypes, IR2 had the best OS outcomes, followed by IR3 and IR1. In terms of anti-angiogenic agents, sunitinib may be appropriate for IR1 patients, while axitinib and pazopanib may be suitable for IR2 patients, and sorafenib for IR3 patients. Additionally, IR1 patients might benefit from anti-CTLA4 therapy. A scoring system called IRscore was defined for individual ccRCC patients. Patients with high IRscore presented a lower response rate to anti-PD-L1 therapy and worse prognostic outcomes. Pan-cancer analysis demonstrated the immunological features and prognostic relevance of the IRscore.

**Conclusion:**

Altogether, characterization of inflammatory response subtypes and IRscore provides a roadmap for patient risk stratification and personalized treatment decisions, not only in ccRCC, but also in pan-cancer.

**Supplementary Information:**

The online version contains supplementary material available at 10.1186/s12920-023-01687-5.

## Introduction

Clear cell renal cell carcinoma (ccRCC) occupies approximately 75% of all renal cancer cases, with a rising global incidence. It is considered an angiogenic and immunogenic tumor [1]. Patients with early-stage ccRCC primarily receive surgical resection, with pharmacological management (anti-angiogenic treatment, immunotherapy, etc.) for those with metastases [2]. About 40% of resected ccRCCs will relapse and develop metastasis [3]. Additionally, rapid resistance to antiangiogenic drugs and low response rates to immune checkpoint blockade (ICB) necessitate novel strategies [4].

Tumors are infiltrated by various immune and non-immune cell types, which possess pro- and anti-tumor effects [5]. The balance of the mediators and cellular effectors of inflammatory response exerts an important role in controlling tumor progression and efficacy of ICB [6]. Therefore, manipulation of the inflammatory response is an attractive approach to enhance ICB efficacy when combined with cancer treatment that can exert immunostimulatory effects, or via directly mitigating pro-tumor inflammation [7]. For instance, anti-inflammatory agents (COX2 inhibitor, EP2-4 PGE2 receptor antagonist, etc.) can remodel the tumor immune landscape to improve tumor immunogenicity and efficacy of ICB [8]. Photothermal therapy combined with nonsteroidal anti-inflammatory drug Aspisol mitigates immunosuppression and enhances anti-cancer treatment [9]. Cytokine as well as immune-checkpoint inhibitors are acceptable therapeutic options for ccRCC, demonstrating that management of inflammatory response is crucial for treating ccRCC [10]. Notably, chronic inflammation is remarkably linked to ccRCC progression, and local and systemic inflammation are usually found in advanced-stage patients, providing an opportunity to target the inflammatory response to improve survival outcomes [11].

Herein, a systematic analysis of inflammatory response features was conducted in large-scale ccRCC cohorts to characterize the inflammatory response-based categorization and define an IRscore system for refining risk stratification and personalized therapy in ccRCC.

## Materials and methods

### Patients and datasets

This study retrospectively analyzed the transcriptome profiling and clinical parameters of ccRCC patients from The Cancer Genome Atlas (TCGA)-kidney renal clear cell carcinoma (KIRC) (*n* = 537), E-MATB-1980 (*n* = 101) and International Cancer Genome Consortium (ICGC) (*n* = 91). Supplementary Table 1 summarized the clinical features of each dataset. Somatic mutation, copy number variation (CNV), DNA methylation and microRNA (miRNA) expression data of ccRCC patients were acquired from TCGA. We gathered two ICB cohorts (IMvigor210 (*n* = 298) [12] and Liu et al. (*n* = 121) [13]), including expression profiles, immunotherapeutic response, and survival information. The transcriptome profiling and prognostic information of pan-cancer were obtained from TCGA.

### Collection of inflammatory response genes

Totally, 200 genes defining inflammatory response were acquired from “HALLMARK_INFLAMMATORY_RESPONSE” gene set of the Molecular Signatures Database (MSigDB; http://www.broadinstitute.org/msigdb) (Supplementary Table 2) [14]. Protein–protein interactions were predicted through the STRING online database [15]. The associations between inflammatory response genes and overall survival (OS) were evaluated via univariate-cox regression analysis. Hazard ratio (HR) and *p*-value were calculated, respectively. The above information combined with the genomic position was visualized in circos track plots using RCircos package [16].

### Discovery of inflammatory response subtypes

Prognosis-related inflammatory response genes were utilized for unsupervised clustering analysis through ConsensusClusterPlus package [17]. The reliability and stability of clusters was further verified through cumulative distribution function (CDF) curves and tracking plots.

### Analysis of immunological features

Common immune checkpoint molecules (CD274, PDCD1, CD247, PDCD1LG2, CTLA4, TNFRSF9, and TNFRSF4) were measured at the transcription level. The relative abundance of 22 immune cell types and two stromal cell types was estimated via single-sample gene set enrichment analysis (ssGSEA) computational method [18]. Additionally, the fractions of infiltrating immune and stromal cells were quantified utilizing ESTIMATE method [19]. Tumor immunogenicity factors comprising tumor mutation burden (TMB), microsatellite instability (MSI), aneuploidy score, cancer testis antigen (CTA) score, homologous recombination defects, intratumor heterogeneity, mRNA expression-based stemness index (mRNAsi), and single nucleotide variants (SNV) neoantigens were collected from TCGA and previously published literature. Based on the gene sets of steps within cancer-immunity cycle [20], activity of each step was computed via Gene Set Variation Analysis (GSVA) [18].

### Functional enrichment analysis

Annotated gene sets (“h.all.v7.4.entrez.gmt” and “c2.cp.kegg.v7.5.1”) of hallmark and Kyoto Encyclopedia of Genes and Genomes (KEGG) pathways were acquired from the MSigDB [14], and pathway activity was estimated with GSVA [18]. Gene ontology (GO) and KEGG pathway enrichment analysis was executed using the ClusterProfiler R package with a threshold of false discovery rate (FDR) < 0.05 [21].

### Genomic mutation analysis

Somatic mutation data with mutation annotation format was utilized for somatic mutation analysis via maftools package [22]. The mutational OncoPrint plots were generated utilizing ComplexHeatmap package [23]. Significant copy-number amplifications and deletions were evaluated through GISTIC2.0 based on the criteria of FDR < 0.25 [24]. Fraction genome altered (FGA), fraction genome gain (FGG), and fraction genome loss (FGL) of each ccRCC sample were calculated, respectively [25].

### External verification of inflammatory response-based categorization

With a threshold of adjusted *p* < 0.05, unique up- or down-regulated genes in each inflammatory response subtype were selected by comparing the other subtypes using the limma computational method [26]. The top 200 down and up-regulated genes were regarded as biomarkers of each inflammatory response subtype. The inflammatory response-based categorization was externally verified in two ccRCC datasets (E-MATB-1980 and ICGC) and two ICB datasets (IMvigor210 and Liu et al.) via nearest template prediction (NTP) algorithm [27].

### Subclass mapping (SubMap)

The expression similarity between groups was estimated through SubMap computational approach [28]. The degree of commonality was computed utilizing GSEA. Bonferroni correction, with a *p*-value of < 0.05, suggested a significant similarity between the groups.

### Drug sensitivity analysis

We collected drug sensitivity data of cancer cell lines from the GDSC [29], CTRP [30], as well as PRISM [31] databases. The GDSC comprises the semi-inhibitory concentration (IC50) values. Additionally, the CTRP and PRISM databases include the area under the curve (AUC) data. The lower the IC50 or AUC value, the higher the drug sensitivity. Transcriptome profiles of CCLs were also obtained from the Cancer Cell Line Encyclopedia [32]. The IC50 or AUC value of a specific compound was computed via pRRophetic package [33].

### Multi-omics analysis of immunomodulators

Multi-omics features (covering transcriptome, methylation and CNV) of 75 immunomodulators (Supplementary Table 3) in each ccRCC sample were analyzed in each ccRCC sample [34].

### Co-expression analysis

Weighted gene co-expression network analysis (WGCNA) was utilized to establish a scale-free co-expression network [35]. Briefly, the similarity matrix was generated via computing the Pearson correlation between genes based on the transcriptome profiling. Next, the optimal soft threshold β was selected for ensuring a scale-free co-expression network. The similarity matrix was transformed to an adjacency matrix, followed by conversion of a topological matrix. Average linkage hierarchical clustering was established for identifying a gene dendrogram, and the co-expression modules were clustered with dynamic tree cut method. A gene module that presented the strongest correlation to inflammatory response subtypes was determined through Pearson correlation test, and the genes in this module were regarded as inflammatory response-related genes, which were further validated through gene significance and module membership.

### Generation of an inflammatory response-related prognostic signature

Inflammatory response-related genes were utilized for least absolute shrinkage and selection operator (LASSO) analysis via glmnet package [36]. Variables with nonzero coefficients were selected through tenfold cross-validation. Inflammatory response-based score (IRscore) was generated based on the regression coefficients derived from multivariable-cox regression analysis for variables and their expression in TCGA-KIRC dataset. The IRscore equation was as follows: $\mathrm{IRscore}=\sum_n\mathrm{coefficientofgene}(\mathrm n)\ast\mathrm{expressionofgene}(\mathrm n)$. All patients were classified as low and high IRscore groups using the median IRscore. The repeatability of the IRscore was externally verified in the E-MATB-1980 dataset.

### Nomogram generation

Uni- and multivariate-cox regression analyses on the IRscore and clinicopathological parameters were executed to select independent prognostic factors using survival package. These were then utilized to generate a nomogram, which was plotted utilizing rms package. The nomogram-predicted and actual OS probabilities were visualized into calibration curves. Concordance index (C-index) was computed to appraise the predictive performance of survival with survConcordance package. Clinical benefits from each variable were evaluated through decision curve analysis.

### Analysis of post-transcriptional mechanisms

Aberrant miRNAs were selected through comparing low and high IRscore groups in accordance with |fold change|> 1.5 and *p* < 0.05. Next, targeted pathways were enriched via KEGG pathway enrichment analysis.

### Statistical analysis

All statistical analyses were achieved using R software. Principal component analysis was conducted to visualize the dissimilarity between groups, and the proportions of explained variances were calculated. Kaplan–Meier curves of OS with log-rank test were implemented via survminer package. Time-dependent receiver operating characteristic (ROC) curves were drawn utilizing timeROC package. Categorical variables between groups were compared via chi-square test. Continuous variables between two groups were compared utilizing Student’s t-test or Wilcoxon rank sum test, with analysis of variance or Kruskal–Wallis test for three or more groups. Pearson or Spearman correlation tests were applied for correlation analysis. Statistical significance was set as *p* < 0.05.

## Results

### Characterization of three inflammatory response subtypes with diverse molecular and immunological features and clinical outcomes

**Fig. 1:**
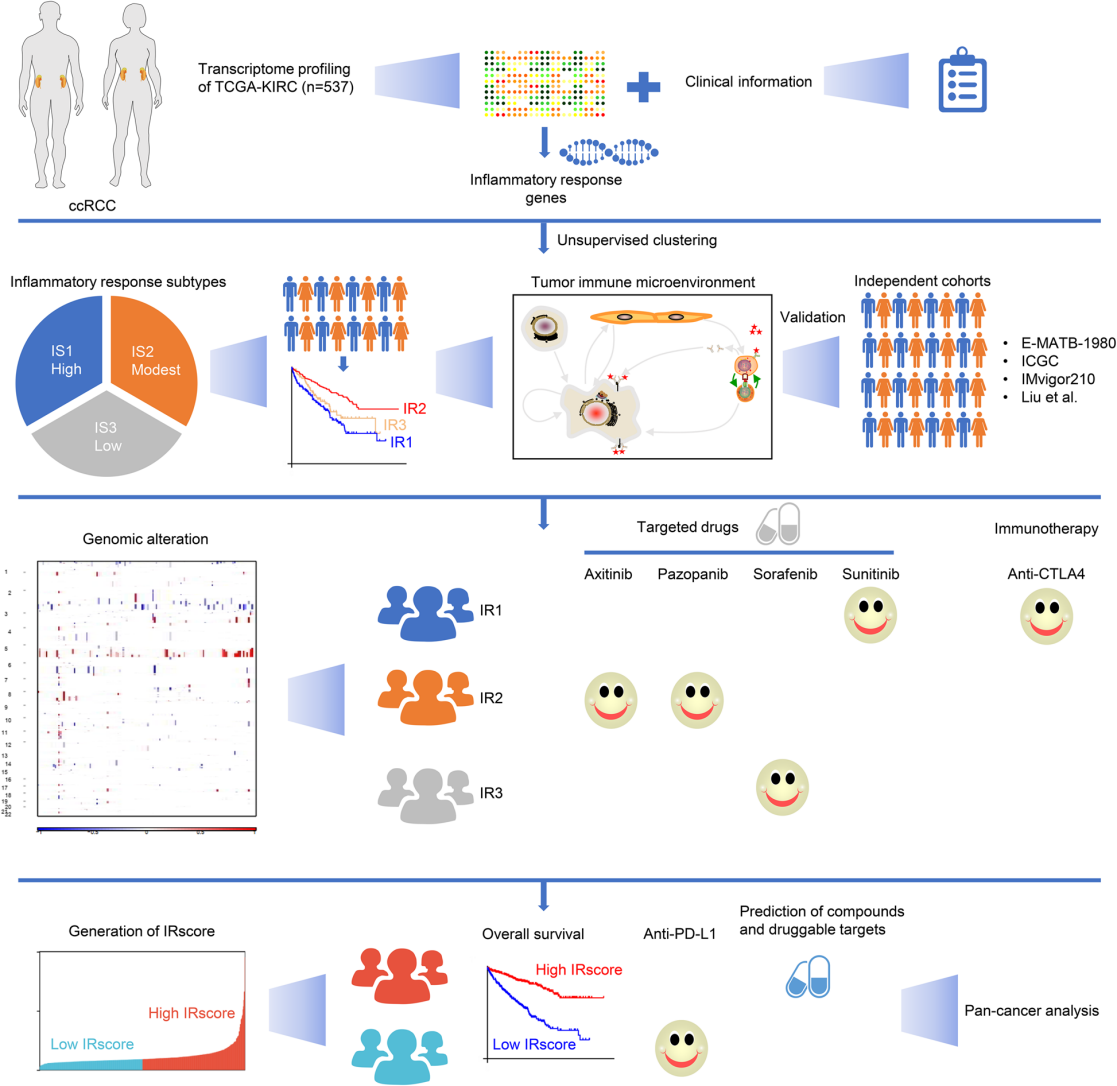
Graphic abstract of the study

**Fig. 2:**
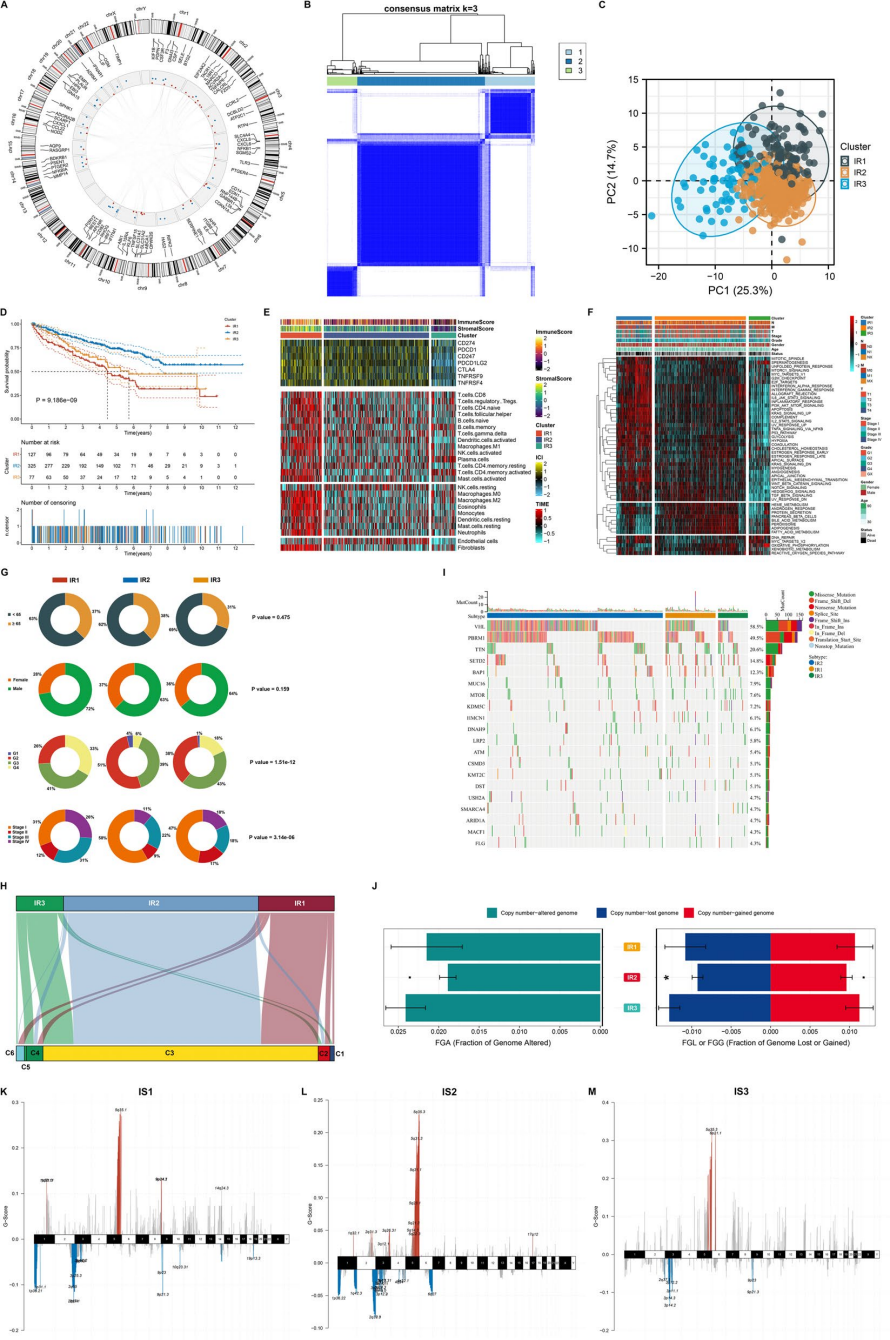
Identification of three inflammatory response subtypes with diverse immunological features, clinical outcomes, and genomic alterations in TCGA-KIRC dataset. **A** Circos track plots illustrate genomic location, interactions, and prognostic relevance of inflammatory response genes. In the inner circle, protein–protein interactions are displayed. Blue dots denote univariate cox regression analysis-derived HR > 1, and red dots denote HR < 1. In the outer circle, genomic location of each inflammatory response gene is shown. **B** Consensus matrix k = 3. Colors from white to blue denote never cluster together to always cluster together. **C** PCA demonstrates the subtype assignments utilizing transcriptome profiles. **D** K-M curves of OS among three inflammatory response subtypes. **E** Transcriptional levels of common immune checkpoint molecules, abundance of immune and stromal cell types, and immune and stromal scores across the three subtypes. **F** Activity of oncogenic hallmark pathways and clinicopathological features in the three subtypes. **G** Differences in clinicopathological parameters among the three subtypes. **H** Sankey diagram illustrates the interactions of inflammatory response subtypes with known immune subtypes. **I** The mutational waterfall among the three inflammatory response subtypes. Mutated genes are ranked by mutational frequency. **J** Differences in the fractions of genome altered, amplification and deletion among the three subtypes. * p < 0.05. (K-M) Detection and comparison of significant copy-number amplifications and deletions across the three subtypes

Figure 1 illustrates a graphic abstract of the study. To systematically dissect the functional roles of inflammatory response in ccRCC, we collected 200 inflammatory response genes. Their genomic locations are illustrated in Fig. 2A. Among these inflammatory response genes, 41 acted as protective factors, with 37 as risk factors (Supplementary Table 4), indicating the prognostic implications of inflammatory response in ccRCC. At the protein level, notable interactions were observed. By unsupervised clustering analysis, we estimated the number of inflammatory response-based classes in TCGA-KIRC dataset. As illustrated in Fig. 2B, ccRCC samples were clearly clustered into three subtypes, named IS1 (high inflammatory response), IS2 (modest inflammatory response), and IS3 (low inflammatory response). When k = 3, the CDF reached an approximate maximum, demonstrating that the categorization was reliable (Figure S1A, B). Moreover, according to tracking plots, the categorization under k = 3 was stable (Figure S1C). PCA also demonstrated that the three subtypes were well distinguished (Fig. 2C). Next, we focused on the difference in OS among the three subtypes. IR2 had the best OS outcomes, followed by IR3 and IR1 (Fig. 2D). Each inflammatory response subtype had unique immunological features. The highest expression of immune checkpoint molecules and the highest abundance of immune and stromal cells were found in IR1, followed by IR2 and IR3 (Fig. 2E). Most oncogenic hallmark pathways presented the highest activity in IR1, contributing to the poorest OS outcomes (Fig. 2F). Analysis of clinicopathological features showed the highest proportions of advanced grade and stage in IR1, followed by IR3 and IR2, partially explaining the difference in OS among the three inflammatory response subtypes (Fig. 2G). More importantly, this inflammatory response subtypes were independent of known immune subtypes (Fig. 2H).

### Genomic features of the three inflammatory response subtypes

To explore the genomic alterations of the three inflammatory response subtypes, we visualized the somatic and CVN frequency of ccRCC patients in TCGA-KIRC dataset. Overall, VHL (58.5%) followed by PBRM1 (49.5%) occurred the highest frequency of mutations in ccRCC (Fig. 2I). The notable differences in mutated VHL and PBRM1 were observed among the three inflammatory response subtypes, with the highest frequency in IR2 (53.1% and 48.8%), followed by IR1 (41.7% and 27.8%) and IR3 (26.7% and 22.2%) (Table 1). Next, this study evaluated the differences in FGA, FGG, and FGL among the three subtypes. Consequently, the highest FGA, FGG, and FGL occurred in IR3, followed by IR1 and the lowest in IR2 (Fig. 2J). This indicated that increased copy-number amplifications and deletions may contribute to low inflammatory response in ccRCC. Follow removal of germline CNVs, significant amplifications and deletions were measured according to FDR < 0.25. We found more regions were altered in IR2 (Fig. 2K-M).

**Table 1:** The differences in mutational frequencies among the three inflammatory response subtypes

Mutated gene	TMB	IR1	IR2	IR3	P	Adjusted p
BAP1	34 (10%)	13 (18.1%)	17 (8.0%)	4 (8.9%)	5.53E-02	1.52E-01
VHL	155 (47%)	30 (41.7%)	113 (53.1%)	12 (26.7%)	2.97E-03	1.63E-02
DST	25 (8%)	6 (8.3%)	14 (6.6%)	5 (11.1%)	5.02E-01	7.30E-01
SETD2	39 (12%)	8 (11.1%)	24 (11.3%)	7 (15.6%)	6.73E-01	8.23E-01
MUC16	23 (7%)	7 (9.7%)	14 (6.6%)	2 (4.4%)	5.31E-01	7.30E-01
DNAH9	17 (5%)	3 (4.2%)	11 (5.2%)	3 (6.7%)	8.68E-01	8.68E-01
LRP2	17 (5%)	3 (4.2%)	11 (5.2%)	3 (6.7%)	8.68E-01	8.68E-01
KDM5C	18 (6%)	2 (2.8%)	12 (5.6%)	4 (8.9%)	3.61E-01	7.30E-01
PBRM1	134 (41%)	20 (27.8%)	104 (48.8%)	10 (22.2%)	1.64E-04	1.80E-03
MTOR	23 (7%)	5 (6.9%)	11 (5.2%)	7 (15.6%)	5.33E-02	1.52E-01
TTN	68 (21%)	11 (15.3%)	47 (22.1%)	10 (22.2%)	4.68E-01	7.30E-01

### Validation of the high reproducibility of the inflammatory response-based categorization in multiple datasets

**Fig. 3:**
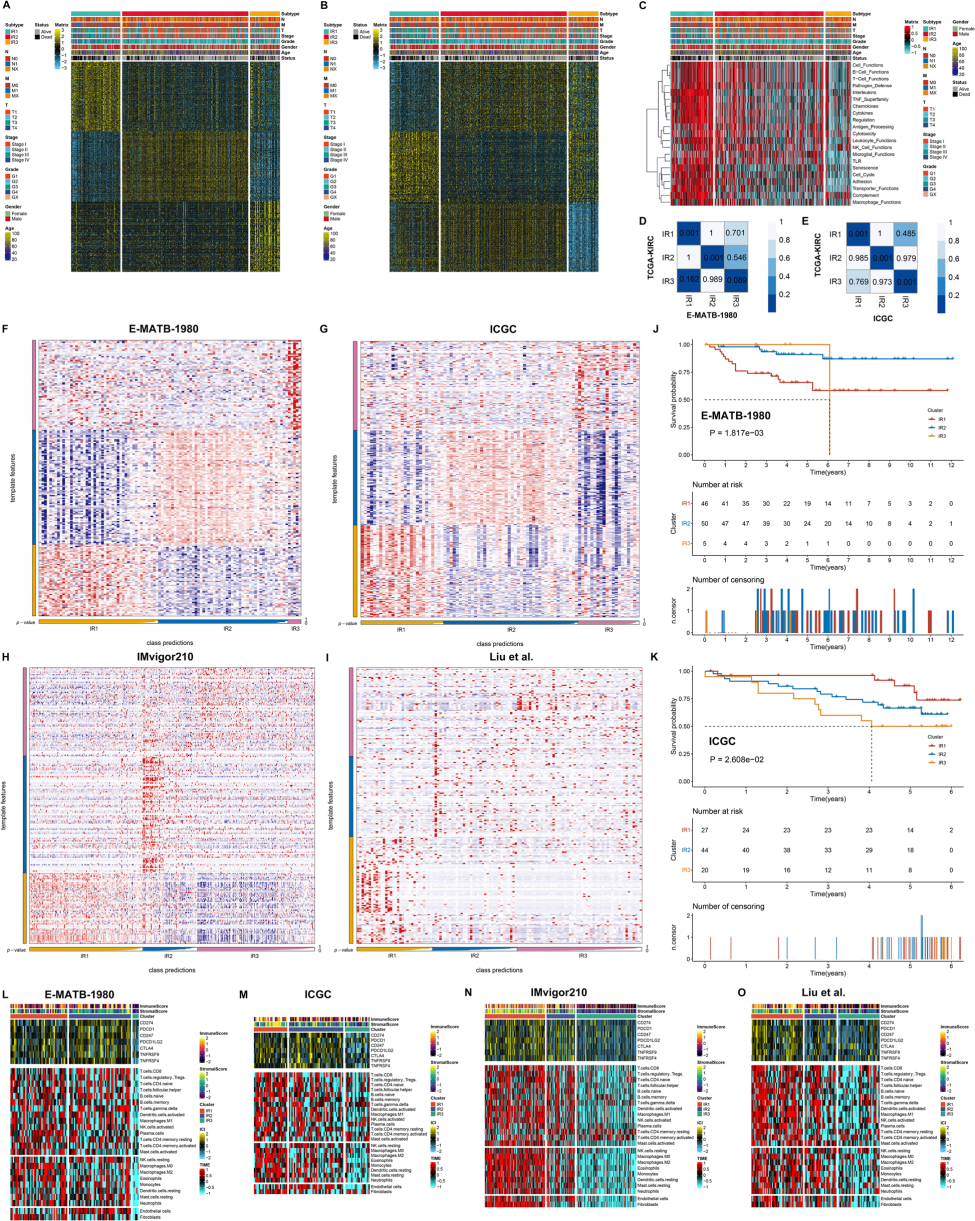
Validation of the high reproducibility of the inflammatory response classification in multiple datasets. **A**, **B** Up- and down-regulated biomarkers of each inflammatory response subtype. **C** Activity of KEGG pathways based on biomarkers. **D** SubMap analysis proves the similarities in inflammatory response subtypes between TCGA-KIRC and E-MATB-1980 datasets. **E** SubMap analysis proves the similarities in inflammatory response subtypes between TCGA-KIRC and ICGC datasets. **F-I** Transcriptome profiling of the template features across inflammatory response subtypes in the E-MATB-1980, ICGC, IMvigor210 and Liu et al. datasets. **J**, **K** K-M curves of OS among subtypes in the E-MATB-1980, and ICGC datasets. **L-O** Validation of transcriptional levels of immune checkpoints, abundance of immune and stromal cells, and immune and stromal scores across subtypes in the E-MATB-1980, ICGC, IMvigor210 and Liu et al. datasets

The top 200 unique up- and down-regulated genes were selected as biomarkers of each inflammatory response subtype (Fig. 3A, B; Supplementary Table 5). Next, KEGG pathways involved in these biomarkers were analyzed. Immunological functions of B cells, T cells, NK cells, and macrophages, etc., interleukins, chemokines, cytokines, complement, etc. presented the highest activity in IR1, indicating high inflammatory response; IR2 was next, and IR3 was the lowest (Fig. 3C). The inflammatory response-based categorization was applied in the E-MATB-1980 and ICGC datasets. The stability and robustness of inflammatory response subtypes was further verified through SubMap and NTP analyses. SubMap results demonstrated that inflammatory response subtypes in TCGA-KIRC dataset were highly similar to those in the E-MATB-1980 and ICGC datasets (Fig. 3D, E). Additionally, NTP analysis revealed that the three inflammatory response subtypes displayed high reproducibility in the E-MATB-1980 and ICGC datasets, respectively (Fig. 3F, G). Similarly, in terms of ICB, we applied the inflammatory response-based categorization to two ICB cohorts. The high reproducibility of this categorization was proven in IMvigor210 and Liu et al. cohorts (Fig. 3H, I). The remarkable differences in OS outcomes among the three inflammatory response subtypes were confirmed in the E-MATB-1980 and ICGC datasets (Fig. 3J, K). We also observed the differences in immune checkpoint molecules and immune and stromal cells in the E-MATB-1980, ICGC, IMvigor210 and Liu et al. datasets (Fig. 3L-O). Hence, the inflammatory response-based categorization was reproducible and stable both in ccRCC and immunotherapy patients.

### Targeted drug resistance of inflammatory response subtypes

**Fig. 4:**
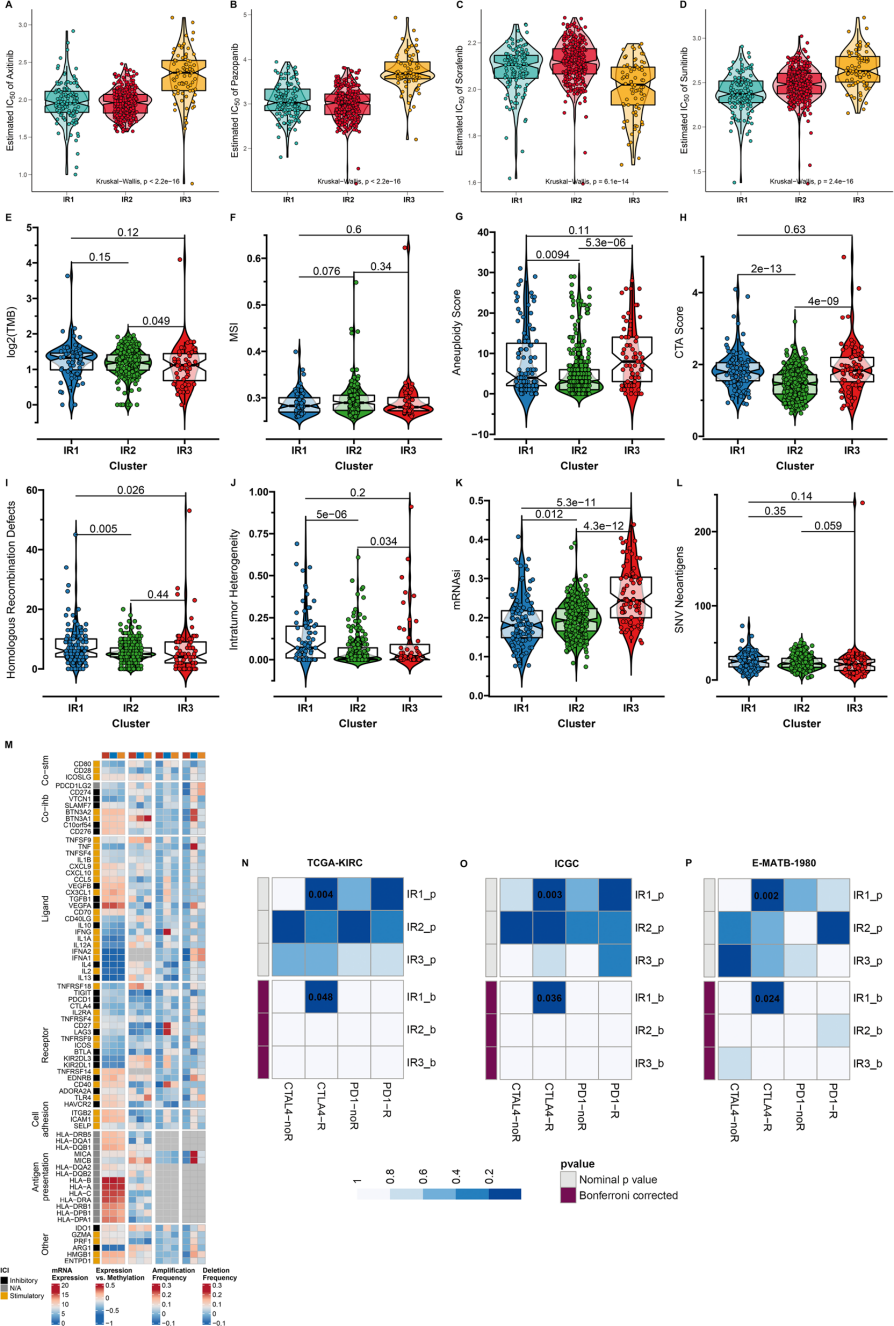
Differences in therapeutic responses and tumor immunogenicity among inflammatory response subtypes. **A**-**D** Comparison of estimated IC50 values of axitinib, pazopanib, sorafenib, and sunitinib among the three inflammatory response subtypes. **E**-**L** Differences in tumor immunogenicity factors including TMB, MSI, aneuploidy score, CTA score, homologous recombination defects, intratumor heterogeneity, mRNAsi, and SNV neoantigens among the subtypes. **M** Landscape of transcriptional levels, methylation, copy-number amplification, and deletion of immunomodulators across the three subtypes. **N**-**P** SubMap analysis demonstrates the similarities in expression patterns between inflammatory response subtypes and responses to anti-PD-1 and anti-CTLA4 in TCGA-KIRC, ICGC and E-MATB-1980 datasets

Small molecule vascular endothelial growth factor receptor (VEGFR) 1/2/3 inhibitors (axitinib [37], pazopanib [38], sorafenib [39], sunitinib [40], etc.) have gained the approval for treating ccRCC. Resistance to these anti-angiogenic agents is always unavoidable. We observed that IR1 displayed the highest sensitivity to sunitinib; IR2 presented the highest resistance to sorafenib, with the highest sensitivity to axitinib and pazopanib; IR3 presented the highest resistance to axitinib, pazopanib and sunitinib, with the highest sensitivity to sorafenib (Fig. 4A-D). Above findings indicated that sunitinib might be suitable for IR1 patients, axitinib and pazopanib for IR2, and sorafenib for IR3.

### Tumor immunogenicity features and ICB efficacy of inflammatory response subtypes

Tumor immunogenicity factors were measured in TCGA-KIRC dataset. No significant differences observed in TMB, MSI, SNV neoantigens between the inflammatory response subtypes; IR2 presented lower aneuploidy score, and CTA score, with higher homologous recombination defects, and intratumor heterogeneity in IR1 (Fig. 4E-L). Also, mRNAsi that reflects stemness had the highest score in IR3, followed by IR2, and the lowest in IR1. Next, the heterogeneity in mRNA expression, methylation and CNVs of immunomodulators was observed among the three inflammatory response subtypes (Fig. 4M). SubMap analysis indicated that IR1 had the high similarity to response to anti-CTLA4 therapy at the transcriptome profiling, which was observed in TCGA-KIRC, ICGC and E-MATB-1980 three datasets (Fig. 4N-P). Hence, IR1 patients might benefit from anti-CTLA4 therapy.

### Recognition of inflammatory response-related genes

**Fig. 5:**
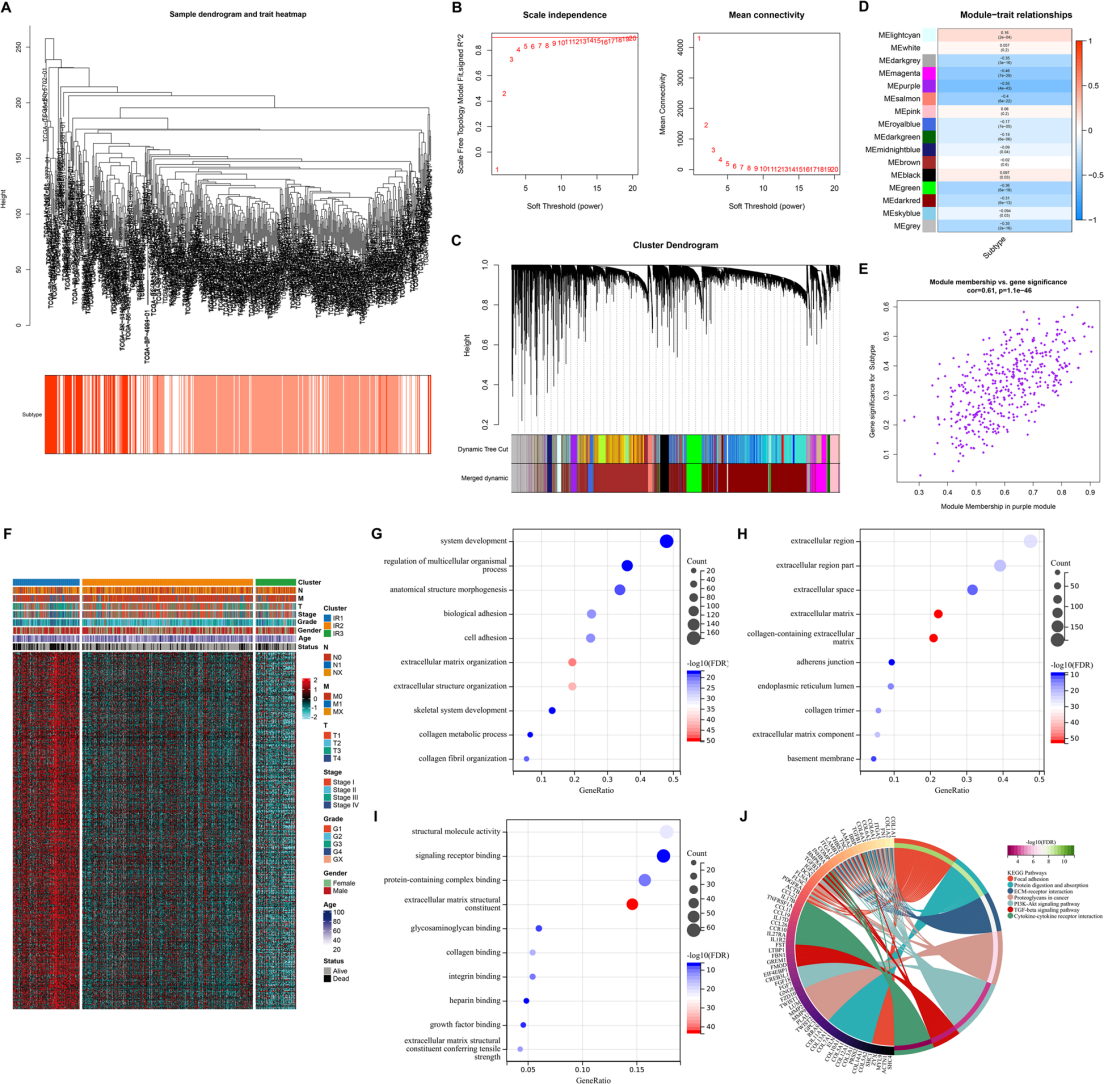
Recognition of inflammatory response-related genes in TCGA-KIRC dataset. **A** Sample dendrogram and heatmap of inflammatory response subtypes. **B** Scale independence together with mean connectivity under distinct soft threshold β values. **C** Cluster dendrogram of co-expression modules. **D** Relationships of co-expression modules with inflammatory response subtypes. **E** Module membership of purple module versus gene significance of inflammatory response subtypes. **F** Transcriptome profiling of inflammatory response-related genes in the three inflammatory response subtypes. **G-J** GO and KEGG enrichment results of inflammatory response-related genes

WGCNA was conducted with transcriptome profiles and inflammatory response subtypes to establish a scale-free co-expression network with the soft threshold β = 20 (Fig. 5A, B). After clustering the genes with similar expression patterns into one module, sixteen co-expression modules were established (Fig. 5C). Purple module presented the strongest correlation to inflammatory response subtypes (Fig. 5D). Genes in this module (*n* = 445) were regarded as inflammatory response-related genes (Fig. 5E; Supplementary Table 6). They showed the highest expression in IR1, followed by IR2, and the lowest in IR3 (Fig. 5F), indicating their inflammatory response relevance. As demonstrated by functional enrichment results, inflammatory response-related pathways (TGF-β, cytokine, etc.) were remarkably enriched by inflammatory response-related genes (Fig. 5G-J).

### Generation and verification of an inflammatory response-based scoring system for ccRCC

**Fig. 6:**
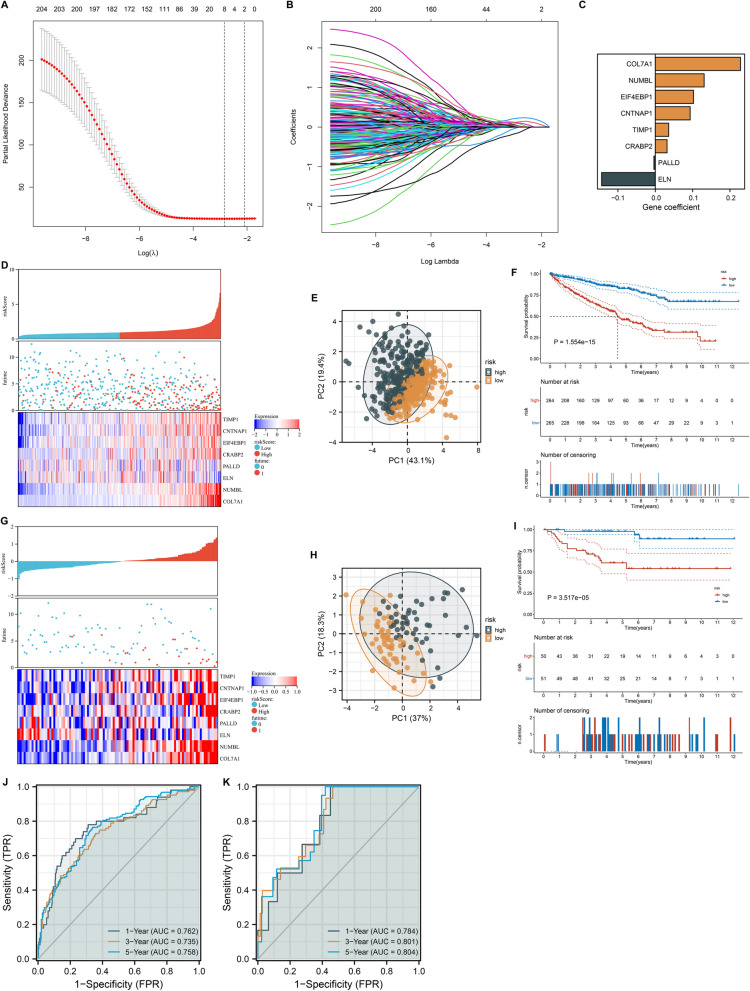
Generation and verification of an inflammatory response-based scoring system for ccRCC. **A, B** LASSO Cox regression analysis for selecting the most robust prognostic inflammatory response-related genes. **C** An ensemble of identified eight genes with individual LASSO coefficients. **D** Distribution of IRscore, survival time, and mRNA expression of each gene in TCGA-KIRC dataset. **E** PCA plots visualize the dissimilarity of low and high IRscore groups in TCGA-KIRC dataset. **F** K-M curves of OS between groups in TCGA-KIRC dataset. **G** Validation of distribution of IRscore, survival time, and mRNA expression of each gene in the E-MATB-1980 dataset. **H** Validation of the dissimilarity of low and high IRscore groups in the E-MATB-1980 dataset. **I** Validation of the OS difference between groups in the E-MATB-1980 dataset. **J, K** ROC curves demonstrate the accuracy of OS prediction in TCGA-KIRC and E-MATB-1980 datasets

To unveil the potential prognostic implications of inflammatory response-related genes, the optimal prognostic signatures were selected with LASSO approach in TCGA-KIRC dataset (Fig. 6A, B). Based on the minimum lambda, eight inflammatory response-related genes were selected to define the inflammatory response-based scoring with the equation of IRscore = (-0.143745793) * ELN expression + (-0.003594107) * PALLD expression + 0.031521892 * CRABP2 expression + 0.03571109 * TIMP1 expression + 0.09335266 * CNTNAP1 expression + 0.102379987 * EIF4EBP1 expression + 0.13084054 * NUMBL expression + 0.228098316 * COL7A1 expression (Fig. 6C). TCGA-KIRC patients were separated into low and high IRscore subgroups, respectively. High IRscore subgroup presented a higher proportion of dead cases (Fig. 6D). PCA result uncovered the remarkable differences in transcriptome profiling between subgroups (Fig. 6E). Patients with high IRscore presented worse OS outcomes than those with low IRscore (Fig. 6F). The high reproducibility of the IRscore was proven in the E-MATB-1980 dataset (Fig. 6G-I). ROC curves at one-, three- and five-year OS demonstrated the excellent efficacy of the IRscore in ccRCC prognostication both in TCGA-KIRC and E-MATB-1980 datasets (Fig. 6J, K).

### Clinical relevance of the IRscore in ccRCC

**Fig. 7:**
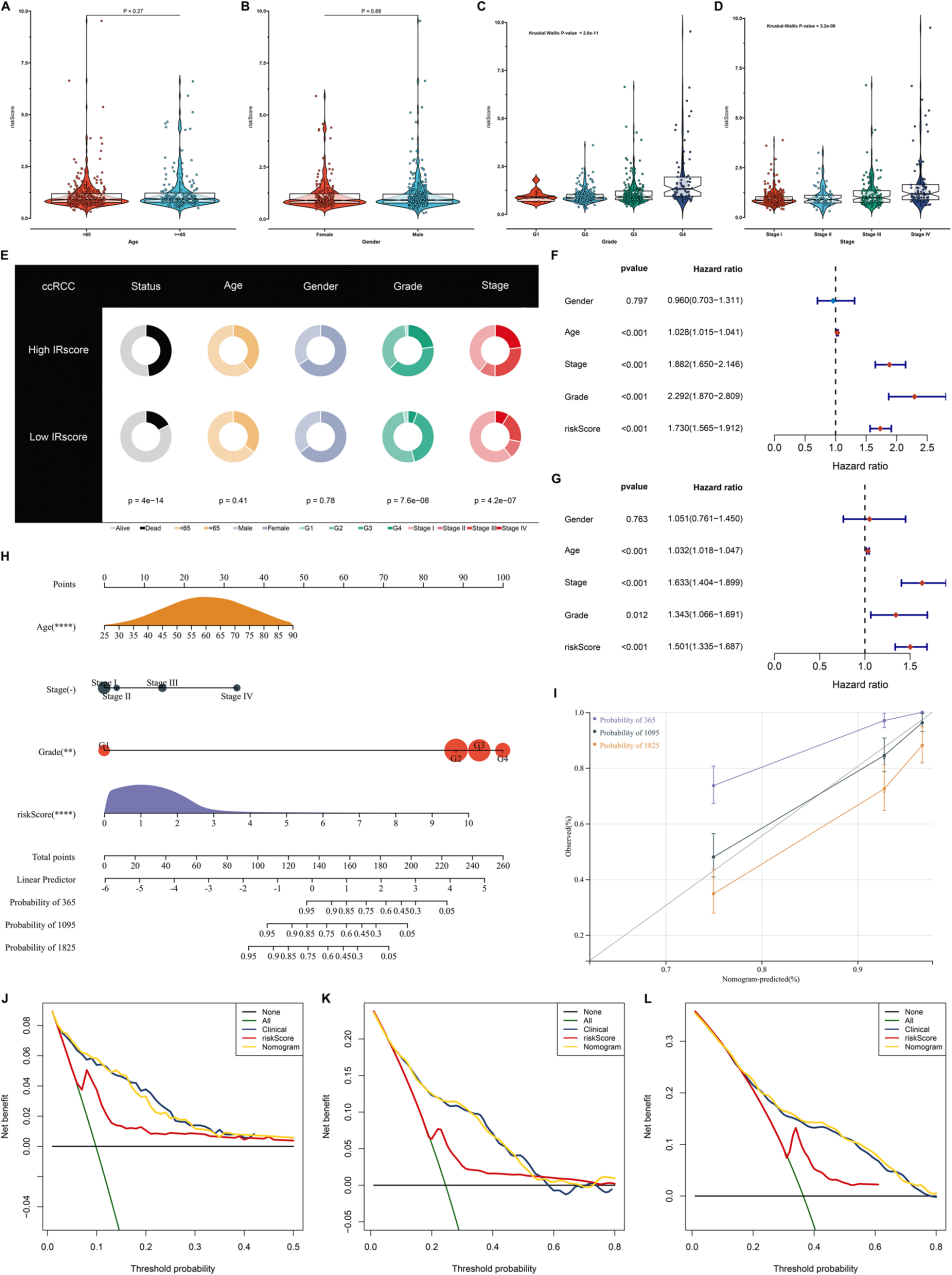
Establishment of an IRscore-based nomogram for individuals in TCGA-KIRC dataset. **A-D** Differences in IRscore between different clinicopathological parameters (age, gender, grade, and stage). **E** Pie plots illustrate the differences in clinicopathological parameters between low and high IRscore groups. **F, G** Uni- and multivariate-cox regression analysis on IRscore and common clinicopathological parameters. **H** Details of the nomogram. **I** Calibration curves show the nomogram-predicted and actual OS probabilities. **J-L** Decision curve analysis graphically illustrates the net benefits of 1-, 3- and 5-year OS from each variable

Next, this study characterized the phenotype correlated to poor prognostic outcomes in high IRscore group. No significant associations of the IRscore with age, and gender were found across ccRCC (Fig. 7A, B). The IRscore gradually increased with grade and stage (Fig. 7C, D). Additionally, high IRscore group had the prominently higher proportions of advanced grade and stage (Fig. 7E). From uni- and multivariate-cox regression analyses, the IRscore and common clinicopathological parameters (age, grade, and stage) acted as independent risk factors of ccRCC prognosis (Fig. 7F, G).

### Establishment of an IRscore-based nomogram for individuals

For providing a readable and quantitative measurement for the IRscore to clinically predict the survival probability, this study generated an integrated nomogram combining the IRscore and other independent clinicopathological parameters (age, stage, and grade), as illustrated in Fig. 7H. C-index was 0.788, indicating the powerful predictive capacity of this nomogram. Calibration curves showed that the nomogram-predicted 1-, 3-, and 5-year OS was nearly coincided with the standard one (Fig. 7I). Additionally, the nomogram achieved the maximum net benefits of survival at 1-, 3- and 5-year OS (Fig. 7J-L).

### Aberrant expression and prognostic implications of the IRscore-derived genes

**Fig. 8:**
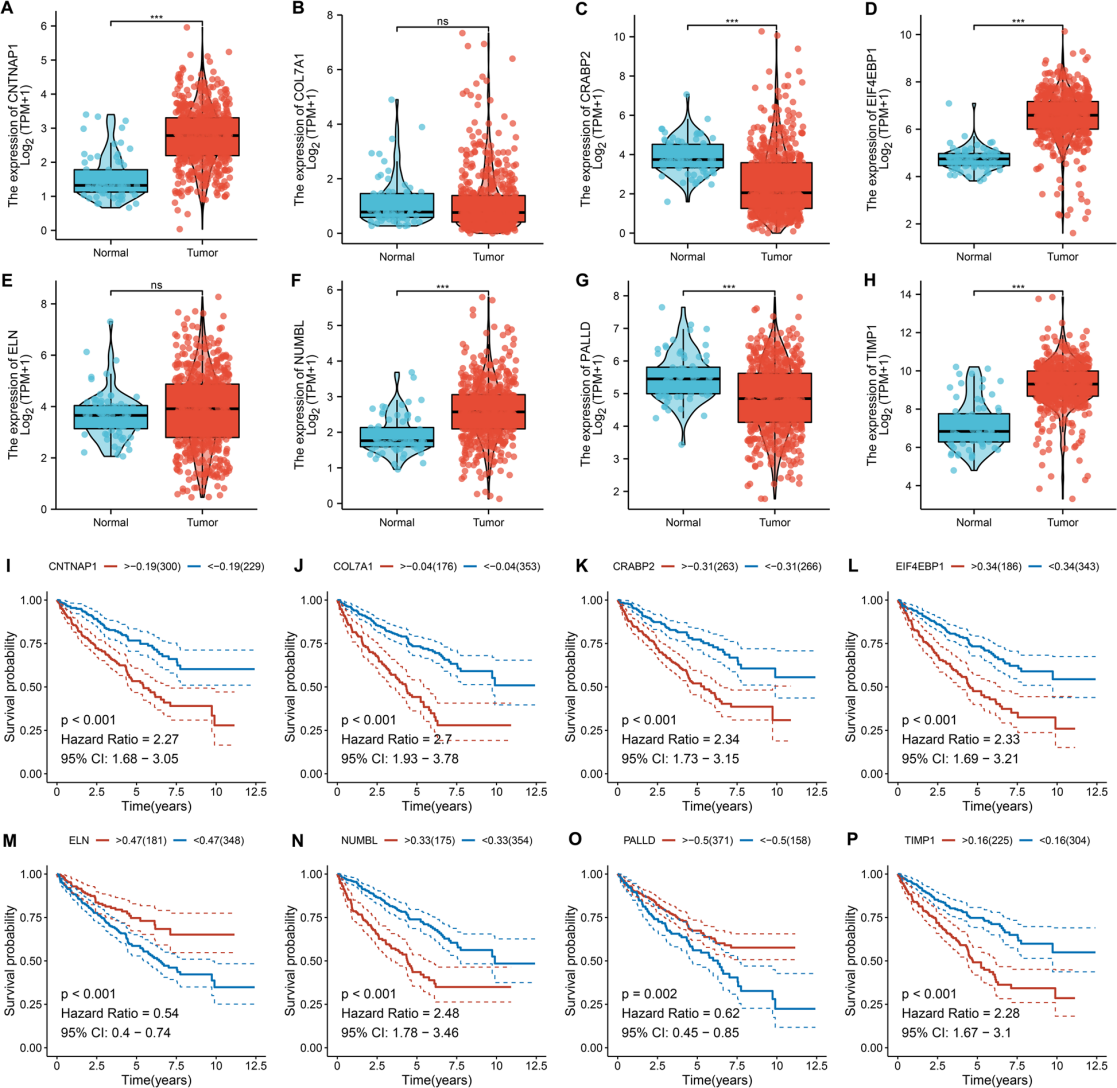
Aberrant expression and prognostic implications of the IRscore-derived genes in TCGA-KIRC dataset. **A-H** Differences in mRNA expression of CNTNAP1, COL7A1, CRABP2, EIF4EBP1, ELN, NUMBL, PALLD and TIMP1 in ccRCC relative to normal tissues. ns: *p* > 0.05; and *** p < 0.001. **I-P** K-M curves of OS between groups separated by the median expression of CNTNAP1, COL7A1, CRABP2, EIF4EBP1, ELN, NUMBL, PALLD and TIMP1

Each gene in the IRscore signature was further analyzed in TCGA-KIRC dataset. Aberrant expression was observed in ccRCC versus normal specimens. CNTNAP1, EIF4EBP1, ELN, NUMBL and TIMP1 were notably up-regulated in ccRCC, with down-regulated COL7A1, CRABP2 and PALLD (Fig. 8A-H). Next, their prognostic value was investigated. Patients with high expression of CNTNAP1, COL7A1, CRABP2, EIF4EBP1, NUMBL and TIMP1 presented worse OS outcomes, with opposite effects for ELN and PALLD (Fig. 8I-P). Moreover, we also investigated the eight genes at protein expression level based on the Human Protein Atlas (HPA) database. EIF4EBP1 showed a relatively high antibody staining level in tumor tissue when compared to normal tissue, while PALLD presented high protein expression level in normal tissue (Figure S2).

### Potential of the IRscore in predicting ICB response

**Fig. 9:**
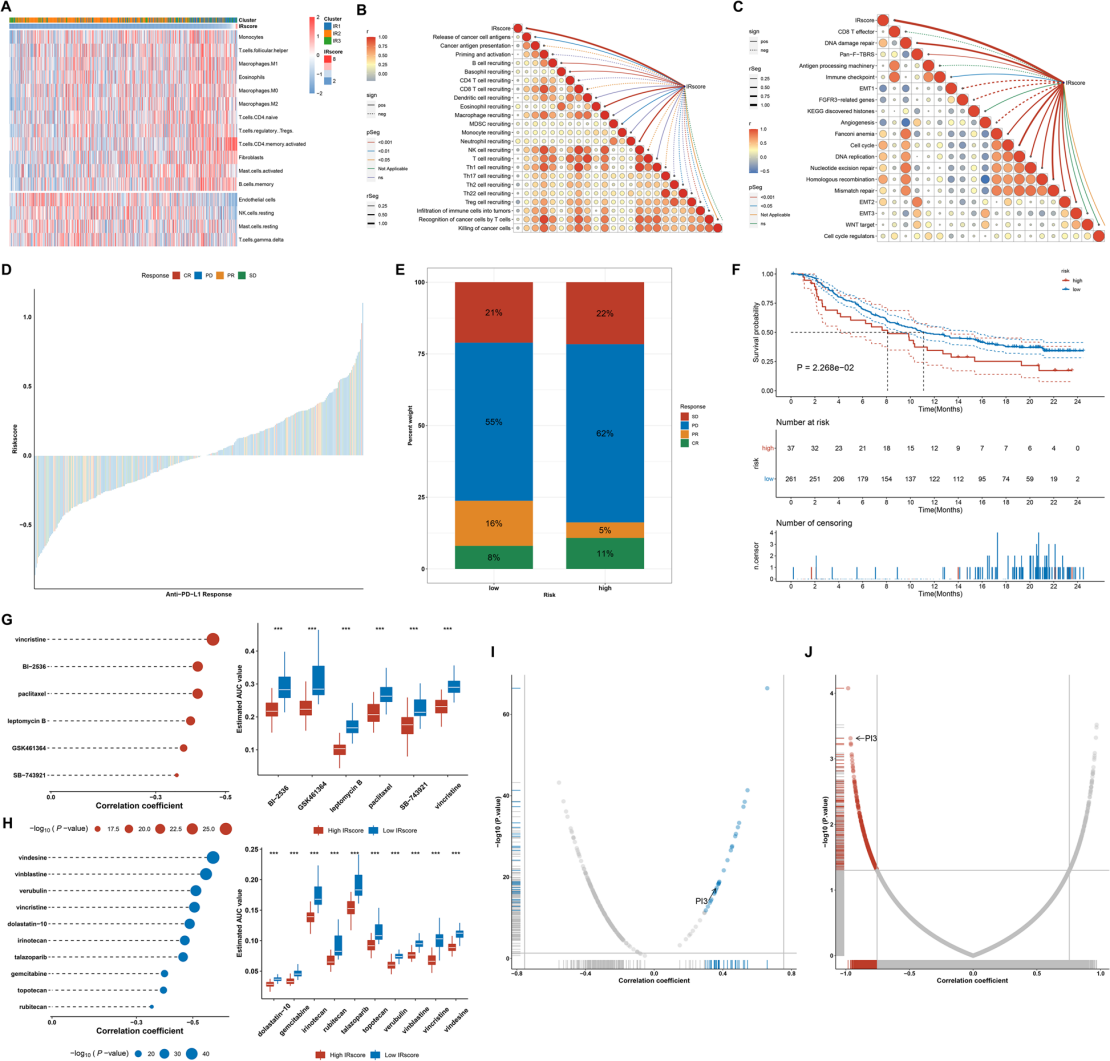
Potential of the IRscore in predicting ICB response and potential compounds and druggable targets. **A** The abundance of immune and stromal cell types across low to high IRscore ccRCC samples in TCGA-KIRC dataset. **B** Associations of the IRscore with the activity of steps within the cancer-immunity cycle in TCGA-KIRC dataset. **C** Associations of the IRscore with the enrichment score of known biological processes in TCGA-KIRC dataset. **D** Distribution of the IRscore across different responses to anti-PD-L1 therapy in the IMvigor210 dataset. **E** Percentage of different therapeutic responses in low and high IRscore groups. **F** K-M curves of OS between groups. **G, H** Correlations between the IRscore and the AUC values of CTRP- or PRISM-derived compounds (left), and differences in the AUC values between low and high IRscore groups. *** p < 0.001. **I, J** Relationships of the IRscore with the protein expression and CERES score of druggable targets. Blue, positive correlation; red, negative correlation

Immune-infiltrating cells within the tumor microenvironment are capable of modulating the tumor phenotype. The high IRscore group presented the higher abundance of immune and stromal cells (Fig. 9A). Cancer immune cycle encompasses a series of steps required for immune-mediated tumor growth control [41]. The IRscore presented the noteworthy relationships with most events of cancer-immunity cycle (Fig. 9B). Further investigation demonstrated that the IRscore was negatively correlated with stromal activation-relevant processes and positively linked to DNA damage repair (Fig. 9C). In light of the immunological relevance of the IRscore, this study further detected the potential of the IRscore in inferring ICB efficacy. On the basis of the IRscore, patients receiving anti-PD-L1 therapy in the IMvigor210 cohort were classified as low and high IRscore subgroups (Fig. 9D). Patients with high IRscore presented worse responses to anti-PD-L1 therapy and more undesirable OS outcomes in comparison to those with low IRscore (Fig. 9E, F). This suggested that the IRscore might be a promising and reliable biomarker of ICB response and clinical outcomes.

### Prediction of promising compounds and druggable targets based on the IRscore

Drug candidates that exhibited higher sensitivity in patients with high IRscore were examined using the CTRP and PRISM datasets. In accordance with correlation coefficient < -0.35 (*p* < 0.05) from association analysis of the IRscore with AUC value, we found six compounds (vincristine, BI-2536, paclitaxel, leptomycin B, GSK461364, and SB-743921) from the CTRP dataset and ten compounds (vindesine, vinblastine, verubulin, vincristine, dolastatin-10, irinotecan, talazoparib, gemcitabine, topotecan and rubitecan) from the PRISM dataset (Fig. 9G, H).

Possible druggable targets that demonstrate significant correlations with the IRscore may hold promise as therapeutic options for patients with high IRscore. Through correlation analysis between the IRscore and protein expression of druggable targets, a total of twenty-eight potential druggable targets were identified, with a correlation coefficient greater than 0.30 (*p* < 0.05) (Fig. 9I; Supplementary Table 7). Additionally, by evaluating the association between the IRscore and CERES score of druggable targets, we identified 213 potential targets with a correlation coefficient less than -0.80 (*p* < 0.05) (Fig. 9J; Supplementary Table 8). Integrating above two analyses, PI3 that encodes an elastase-specific inhibitor was finally identified as promising therapeutic targets, implying that mitigating the function of PI3 in patients with high IRscore might lead to beneficial therapeutic effects.

### Post-transcriptional mechanisms underlying the IRscore

**Fig. 10:**
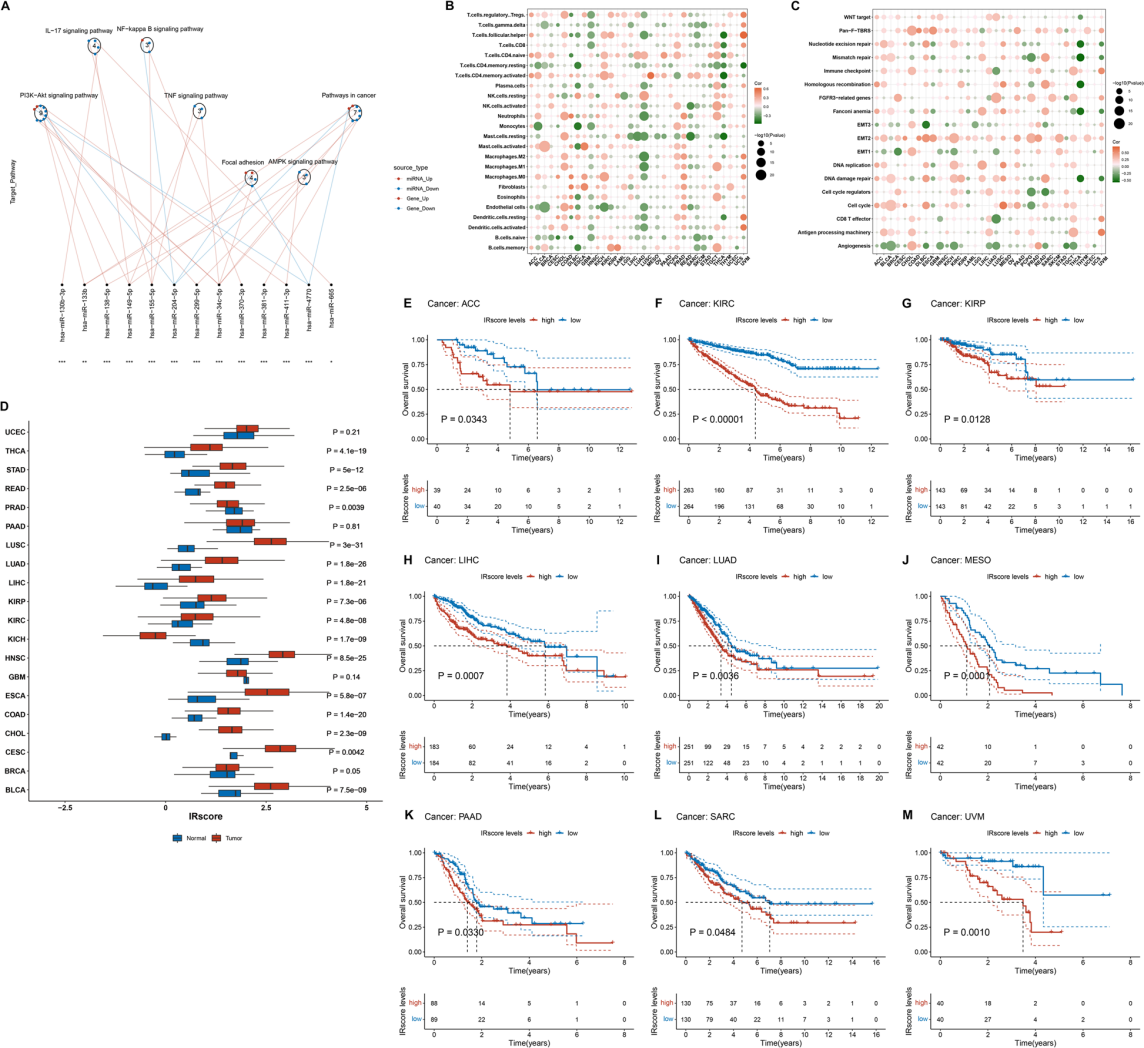
Post-transcriptional mechanisms underlying the IRscore and pan-cancer analysis of immunological features and prognostic relevance. **A** Differences in miRNA-targeted signaling pathways in TCGA-KIRC dataset between low and high IRscore groups. Red dots denote miRNA-targeted mRNAs that are up-regulated in high IRscore group, while blue dots denote miRNA-targeted that are down-regulated in high IRscore group. The circles represent signaling pathways enriched by targeted mRNAs. Red lines indicate up-regulated miRNAs in high IRscore group, while blue lines indicate down-regulated miRNAs in high IRscore group. **B, C** Bubble diagram illustrates the associations of IRscore with the abundance of immune and stromal cells and the activity of known biological processes across pan-cancer. **D** Differences in IRscore between tumors and normal tissues for each cancer type. **E-M** K-M curves of OS between low and high IRscore groups in different cancer types

Next, we assessed the power of the IRscore in interpretating post-transcriptional events. Differences in miRNA expression were analyzed in TCGA-KIRC dataset. Consequently, 177 miRNAs with differential expression were determined between low and high IRscore groups (Supplementary Table 9). Afterwards, we executed functional enrichment analysis of their targeted mRNAs. As illustrated in Fig. 10A, oncogenic pathways (PI3K-Akt, IL-17, NF-ΚB, TNF, AMPK, focal adhesion, pathways in cancer, etc.) were notably enriched, suggesting the IRscore was linked with post-transcriptional events as well as regulation of oncogenic pathways.

### Immunological features and prognostic relevance of the IRscore across pan-cancer

Pan-cancer analysis was conducted to further elucidate the immunological features and prognostic relevance of the IRscore in different cancer types. Notably, the IRscore was significantly correlated to the abundance of most immune and stromal cells across pan-cancer (Fig. 10B). Additionally, we observed the prominent relationships of the IRscore with stromal and immune activation-related pathways in each cancer type (Fig. 10C). Next, we analyzed the differences in the IRscore between tumors and normal tissues. In most cancer types, the higher IRscore was observed in tumors versus normal tissues (Fig. 10D). Moreover, patients with high IRscore presented poorer OS outcomes in comparison to those with low IRscore across multiple cancers (Fig. 10E-M). Above findings demonstrated that the IRscore played essential roles in the tumor immune microenvironment and clinical outcomes across pan-cancer.

## Discussion

Tumor progression and therapeutic response are mediated by inflammatory response, either promoting or mitigating tumor progression, potentially showing opposite effects on treatment outcomes [42]. Inducing acute inflammatory response usually triggers the maturation of dendritic cells as well as antigen presentation, resulting in anti-tumor immunity, while chronic inflammation promotes tumor development and therapeutic resistance [43]. Tumor microenvironment contains diverse inflammatory cells and mediators, and targeting these factors remarkably lowers tumor development, growth and spread [44]. Therefore, the intricate network of the inflammatory response presents potential avenues for the prevention and treatment of ccRCC. However, the clinical research required to substantiate the targeting of cancer-related inflammation and innate immunity in cancer patients is still in its nascent stages [45]. Here, we comprehensively investigated inflammatory response features across ccRCC tumors from multiple datasets, and characterized the inflammatory response-based categorization as well as defined the IRscore system for ccRCC patients.

Three highly reproducible inflammatory response subtypes with distinct molecular, clinical, and immunological features were established in ccRCC. IR2 had the best OS outcomes, followed by IR3 and IR1. Large-scale tumor transcriptome analysis reveals that ccRCC is a highly immune-infiltrating solid tumor, but high immune infiltration is linked with an undesirable prognosis following nephrectomy [46]. Depending upon the balance of immune cell types and signaling within the tumor microenvironment, inflammation can support or suppress tumor progression. Innate immune cell types present high heterogeneity and plasticity, and their phenotypes vary by grading, staging, etc. Immune cells and myofibroblasts are recognized as primary contributors to chronic inflammation [30]. There was widespread heterogeneity in immune and stromal cells among the subtypes, with the highest abundance in IR1, followed by IR2 and IR3.

Tumors develop from normal cells by acquiring genetic alterations that allow them to proliferate uncontrollably. Genomic instability is a hallmark of cancer and shapes the genomic makeup of tumor cells, thus determining their behaviors and therapeutic response [47]. Evidence suggests that genomic instability causes activation of immune response. Notably, VHL and PBRM1 occurred the highest mutated frequency in IR2 (53.1% and 48.8%), followed by IR1 (41.7% and 27.8%) and IR3 (26.7% and 22.2%). We also investigated the CNV heterogeneity among the subtypes. Thus, genomic alterations might correlate to inflammatory response in ccRCC. Due to the relationship between inflammation and tumors, exploiting inflammation appears to be a crucial approach for more effective anti-cancer therapy. Anti-angiogenic agents act not only on endothelial and tumor cells, but also on immune cells [48]. However, the rapid development of resistance has impeded the effective implementation of anti-angiogenic agents in clear cell renal cell carcinoma (ccRCC). In this study, we have identified that sunitinib may be a suitable treatment option for patients with immune response subtype 1 (IR1), while axitinib and pazopanib may be more appropriate for patients with immune response subtype 2 (IR2), and sorafenib for patients with immune response subtype 3 (IR3). Over the past decade, immunotherapy, particularly immune checkpoint blockade (ICB), has had a significant impact on the treatment of ccRCC. However, the therapeutic response rate remains relatively low [49]. Among the three inflammatory response subtypes, IR1 patients may derive benefits from anti-CTLA4 therapy, suggesting that the induction of inflammatory signaling could potentially enhance the efficacy of ICB therapy in ccRCC.

Recently, computational biology achieved much progresses in exploring molecular mechanisms and targets of disease or tumor. For example, Bao et al. proposed a human-specific method [50] and 2-hydr_Ensemble residues’ identification algorithm [51], which provide new perspective in the studies of acetylation process in human body and improve the accuracy of modification residues identification, respectively. In our study, we defined an inflammatory response-based scoring (covering CNTNAP1, COL7A1, CRABP2, EIF4EBP1, ELN, NUMBL, PALLD, and TIMP1) called IRscore for individual ccRCC patients, which enabled to precisely predict patient survival and estimate anti-PD-L1 therapy efficacy. Limited evidence has revealed the implications of a single gene from the IRscore in ccRCC. A bioinformatics study found that CNTNAP1 is up-regulated in ccRCC, and appreciably linked with poor clinical outcomes and immunological properties [52]. Two other bioinformatics studies demonstrated the prognostic relevance of COL7A1 and CRABP2 in ccRCC [53, 54]. Experimental research revealed that EIF4EBP1 facilitates ccRCC cell proliferation and metastasis [55]. Our pan-cancer analysis demonstrated the immunological features and prognostic relevance of the IRscore. Thus, we speculated that inflammatory response served as a synergistic treatment target in ICB not only in ccRCC but also across pan-cancer.

Nonetheless, several limitations exist and should be acknowledged. First, the public datasets cannot provide direct information on the tumor microenvironment. Thus, we only indirectly inferred the abundance of immune and stromal compositions through computational methods. Second, further molecular experiments should be implemented to observe the functional roles of the identified inflammatory response-related genes in ccRCC. Third, although this retrospective study attempted to contain as many ccRCC patients as possible for more religious conclusion, the inflammatory response-based categorization requires prospective validation in independent well-designed clinical trials. Finally, all the result were based on the exited datasets analysis and in vitro and in vivo experiments need to be conducted to further validated these results.

### Supplementary Information

**Additional file 1: Figure S1.** Estimation of the number of inflammatory response-based unsupervised classes in TCGA-KIRC dataset. (A) Consensus CDF curves show consensus distributions for each k. (B) Delta area plots exhibit the relative alteration in area under CDF curve for k relative to k-1. (C) Item tracking plots display the consensus cluster of items at each k. **Figure S2.** Validation of the eight genes on the protein expression level based on HPA database.

**Additional file 2. **The clinical information of TCGA-KIRC, E-MATB-1980, and ICGC datasets.

**Additional file 3.** The list of 200 genes defining inflammatory response.

**Additional file 4.** The gene set of immunomodulators.

**Additional file 5.** Prognosis-related inflammatory response genes.

**Additional file 6.** Biomarkers of three inflammatory response subtypes

**Additional file 7.** Inflammatory response-related genes.

**Additional file 8.** Association between the IRscore and protein expression of druggable targets.

**Additional file 9.** Association between the IRscore and CERES score of druggable targets

**Additional file 10.** MiRNAs with differential expression between low and high IRscore groups.

## Data Availability

The datasets analyzed in this study are available in the TCGA (https://portal.gdc.cancer.gov/), ICGC (https://dcc.icgc.org/), and GEO (https://www.ncbi.nlm.nih.gov/geo/) database.

## Abbreviations

ccRCC: Clear cell renal cell carcinoma
ICB: Immune checkpoint blockade
TCGA: The Cancer Genome Atlas
KIRC: Kidney renal clear cell carcinoma
ICGC: International Cancer Genome Consortium
CNV: Copy number variation
miRNA: MicroRNA
MSigDB: Molecular Signatures Database
OS: Overall survival
HR: Hazard ratio
CDF: Cumulative distribution function
ssGSEA: Single-sample gene set enrichment analysis
TMB: Tumor mutation burden
MSI: Microsatellite instability
CTA: Cancer testis antigen
mRNAsi: MRNA expression-based stemness index
SNV: Single nucleotide variants
GSVA: Gene Set Variation Analysis
KEGG: Kyoto Encyclopedia of Genes and Genomes
GO: Gene ontology
FDR: False discovery rate
FGA: Fraction genome altered
FGG: Fraction genome gain
FGL: Fraction genome loss
NTP: Nearest template prediction
SubMap: Subclass Mapping
IC50: Semi-inhibitory concentration
AUC: Area under the curve
WGCNA: Weighted gene co-expression network analysis
LASSO: Least absolute shrinkage and selection operator
C-index: Concordance index
ROC: Receiver operating characteristic
HPA: Human Protein Atlas

## References

[1] Udayakumar D, Zhang Z, Xi Y (2021). Deciphering intratumoral molecular heterogeneity in clear cell renal cell carcinoma with a radiogenomics platform. Clin Cancer Res.

[2] Bui TO, Dao VT, Nguyen VT (2022). Genomics of clear-cell renal cell carcinoma: a systematic review and meta-analysis. Eur Urol.

[3] Obradovic A, Chowdhury N, Haake SM (2021). Single-cell protein activity analysis identifies recurrence-associated renal tumor macrophages. Cell.

[4] Terry S, Dalban C, Rioux-Leclercq N (2021). Association of AXL and PD-L1 expression with clinical outcomes in patients with advanced renal cell carcinoma treated with PD-1 blockade. Clin Cancer Res.

[5] Kim MC, Borcherding N, Ahmed KK (2021). CD177 modulates the function and homeostasis of tumor-infiltrating regulatory T cells. Nat Commun.

[6] Hsu SK, Li CY, Lin IL (2021). Inflammation-related pyroptosis, a novel programmed cell death pathway, and its crosstalk with immune therapy in cancer treatment. Theranostics.

[7] Morris EC, Neelapu SS, Giavridis T, Sadelain M (2022). Cytokine release syndrome and associated neurotoxicity in cancer immunotherapy. Nat Rev Immunol.

[8] Pelly VS, Moeini A, Roelofsen LM (2021). Anti-inflammatory drugs remodel the tumor immune environment to enhance immune checkpoint blockade efficacy. Cancer Discov.

[9] Zhang D, Yang J, Ye S (2022). Combination of photothermal therapy with anti-inflammation therapy attenuates the inflammation tumor microenvironment and weakens immunosuppression for enhancement antitumor treatment. Small.

[10] Nishida J, Momoi Y, Miyakuni K (2020). Epigenetic remodelling shapes inflammatory renal cancer and neutrophil-dependent metastasis. Nat Cell Biol.

[11] Braun DA, Street K, Burke KP (2021). Progressive immune dysfunction with advancing disease stage in renal cell carcinoma. Cancer Cell.

[12] Mariathasan S, Turley SJ, Nickles D (2018). TGFβ attenuates tumour response to PD-L1 blockade by contributing to exclusion of T cells. Nature.

[13] Liu D, Schilling B, Liu D (2019). Integrative molecular and clinical modeling of clinical outcomes to PD1 blockade in patients with metastatic melanoma. Nat Med.

[14] Liberzon A, Birger C, Thorvaldsdóttir H (2015). The Molecular Signatures Database (MSigDB) hallmark gene set collection. Cell Syst.

[15] Szklarczyk D, Gable AL, Nastou KC (2021). The STRING database in 2021: customizable protein-protein networks, and functional characterization of user-uploaded gene/measurement sets. Nucleic Acids Res.

[16] Zhang H, Meltzer P, Davis S (2013). RCircos: an R package for Circos 2D track plots. BMC Bioinformatics.

[17] Wilkerson MD, Hayes DN (2010). ConsensusClusterPlus: a class discovery tool with confidence assessments and item tracking. Bioinformatics.

[18] Hänzelmann S, Castelo R, Guinney J (2013). GSVA: gene set variation analysis for microarray and RNA-seq data. BMC Bioinformatics.

[19] Yoshihara K, Shahmoradgoli M, Martínez E (2013). Inferring tumour purity and stromal and immune cell admixture from expression data. Nat Commun.

[20] Chen DS, Mellman I (2013). Oncology meets immunology: the cancer-immunity cycle. Immunity.

[21] Yu G, Wang LG, Han Y, He QY (2012). clusterProfiler: an R package for comparing biological themes among gene clusters. OMICS.

[22] Mayakonda A, Lin DC, Assenov Y (2018). Maftools: efficient and comprehensive analysis of somatic variants in cancer. Genome Res.

[23] Gu Z, Eils R, Schlesner M (2016). Complex heatmaps reveal patterns and correlations in multidimensional genomic data. Bioinformatics.

[24] Mermel CH, Schumacher SE, Hill B (2011). GISTIC2.0 facilitates sensitive and confident localization of the targets of focal somatic copy-number alteration in human cancers. Genome Biol.

[25] Moore LE, Jaeger E, Nickerson ML (2012). Genomic copy number alterations in clear cell renal carcinoma: associations with case characteristics and mechanisms of VHL gene inactivation. Oncogenesis.

[26] Ritchie ME, Phipson B, Wu D (2015). limma powers differential expression analyses for RNA-sequencing and microarray studies. Nucleic Acids Res.

[27] Hoshida Y (2010). Nearest template prediction: a single-sample-based flexible class prediction with confidence assessment. PLoS ONE.

[28] Hoshida Y, Brunet JP, Tamayo P (2007). Subclass mapping: identifying common subtypes in independent disease data sets. PLoS ONE.

[29] Yang W, Soares J, Greninger P (2013). Genomics of Drug Sensitivity in Cancer (GDSC): a resource for therapeutic biomarker discovery in cancer cells. Nucleic Acids Res.

[30] Nguyen-Tran HH, Nguyen TN, Chen CY, Hsu T (2021). Endothelial reprogramming stimulated by oncostatin M promotes inflammation and tumorigenesis in VHL-deficient kidney tissue. Cancer Res.

[31] Yu C, Mannan AM, Yvone GM (2016). High-throughput identification of genotype-specific cancer vulnerabilities in mixtures of barcoded tumor cell lines. Nat Biotechnol.

[32] Barretina J, Caponigro G, Stransky N (2012). The Cancer Cell Line Encyclopedia enables predictive modelling of anticancer drug sensitivity. Nature.

[33] Geeleher P, Cox N, Huang RS (2014). pRRophetic: an R package for prediction of clinical chemotherapeutic response from tumor gene expression levels. PLoS ONE.

[34] Liu Z, Zhang Y, Shi C (2021). A novel immune classification reveals distinct immune escape mechanism and genomic alterations: implications for immunotherapy in hepatocellular carcinoma. J Transl Med.

[35] Langfelder P, Horvath S (2008). WGCNA: an R package for weighted correlation network analysis. BMC Bioinformatics.

[36] Engebretsen S, Bohlin J (2019). Statistical predictions with glmnet. Clin. Epigenetics.

[37] Powles T, Plimack ER, Soulières D (2020). Pembrolizumab plus axitinib versus sunitinib monotherapy as first-line treatment of advanced renal cell carcinoma (KEYNOTE-426): extended follow-up from a randomised, open-label, phase 3 trial. Lancet Oncol.

[38] Motzer RJ, Haas NB, Donskov F (2017). Randomized Phase III Trial of Adjuvant Pazopanib Versus Placebo After Nephrectomy in Patients With Localized or Locally Advanced Renal Cell Carcinoma. J Clin Oncol.

[39] Rini BI, Pal SK, Escudier BJ (2020). Tivozanib versus sorafenib in patients with advanced renal cell carcinoma (TIVO-3): a phase 3, multicentre, randomised, controlled, open-label study. Lancet Oncol.

[40] Motzer RJ, Rini BI, McDermott DF (2019). Nivolumab plus ipilimumab versus sunitinib in first-line treatment for advanced renal cell carcinoma: extended follow-up of efficacy and safety results from a randomised, controlled, phase 3 trial. Lancet Oncol.

[41] Somarribas Patterson LF, Vardhana SA (2021). Metabolic regulation of the cancer-immunity cycle. Trends Immunol.

[42] Crusz SM, Balkwill FR (2015). Inflammation and cancer: advances and new agents. Nat Rev Clin Oncol.

[43] Zhao H, Wu L, Yan G (2021). Inflammation and tumor progression: signaling pathways and targeted intervention. Signal Transduct Target Ther.

[44] Nakamura K, Smyth MJ (2020). Myeloid immunosuppression and immune checkpoints in the tumor microenvironment. Cell Mol Immunol.

[45] Maiorino L, Daßler-Plenker J, Sun L, Egeblad M (2022). Innate Immunity and Cancer Pathophysiology. Annu Rev Pathol.

[46] Au L, Hatipoglu E, Robert de Massy M (2021). Determinants of anti-PD-1 response and resistance in clear cell renal cell carcinoma. Cancer Cell.

[47] Chen M, Linstra R, van Vugt M (2022). Genomic instability, inflammatory signaling and response to cancer immunotherapy. Biochim Biophys Acta Rev Cancer.

[48] Haas NB, Manola J, Uzzo RG (2016). Adjuvant sunitinib or sorafenib for high-risk, non-metastatic renal-cell carcinoma (ECOG-ACRIN E2805): a double-blind, placebo-controlled, randomised, phase 3 trial. Lancet.

[49] Motzer RJ, Penkov K, Haanen J (2019). Avelumab plus Axitinib versus Sunitinib for Advanced Renal-Cell Carcinoma. N Engl J Med.

[50] Bao W, Yang B (2024). Protein acetylation sites with complex-valued polynomial model. Front Comput Sci.

[51] Bao W, Yang B, Chen B (2021). 2-hydr_Ensemble: Lysine 2-hydroxyisobutyrylation Identification with Ensemble Method. Chemometr Intell Lab Syst.

[52] Li W, Meng X, Yuan H (2022). M2-polarization-related CNTNAP1 gene might be a novel immunotherapeutic target and biomarker for clear cell renal cell carcinoma. IUBMB Life.

[53] Liao Z, Yao H, Wei J (2021). Development and validation of the prognostic value of the immune-related genes in clear cell renal cell carcinoma. Transl Androl Urol.

[54] He Z, Deng T, Duan X, Zeng G. Profiles of overall survival-related gene expression-based risk signature and their prognostic implications in clear cell renal cell carcinoma. Biosci Rep 2020;40. 10.1042/BSR20200492 PMC7494988 32789468

[55] Gui CP, Liao B, Luo CG (2021). circCHST15 is a novel prognostic biomarker that promotes clear cell renal cell carcinoma cell proliferation and metastasis through the miR-125a-5p/EIF4EBP1 axis. Mol Cancer.

