# Supplementary material for: Inflammatory response-based prognostication and personalized therapy decisions in clear cell renal cell cancer to aid precision oncology
Source: BMC Med Genomics. 2023 Oct 26;16:265. doi: 10.1186/s12920-023-01687-5 (PMC10601329; doi:10.1186/s12920-023-01687-5)

**Figure S1.** Estimation of the number of inflammatory response-based unsupervised classes in TCGA-KIRC dataset. (A) Consensus CDF curves show consensus distributions for each k. (B) Delta area plots exhibit the relative alteration in area under CDF curve for k relative to k-1. (C) Item tracking plots display the consensus cluster of items at each k.


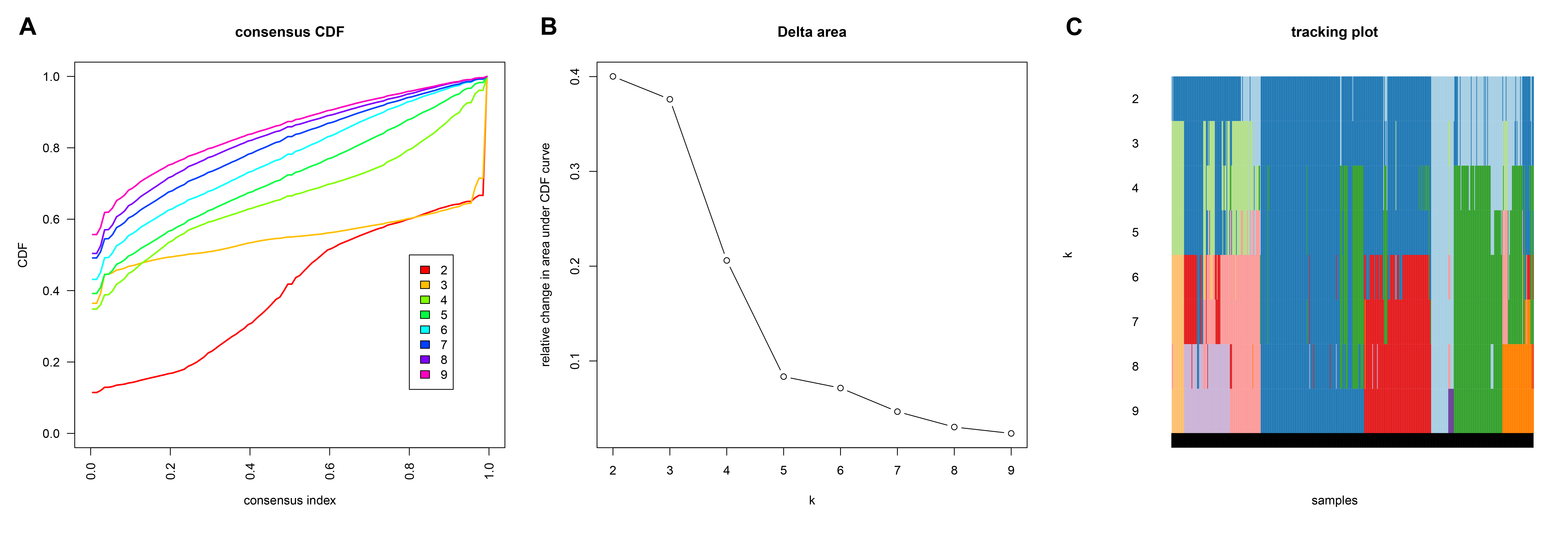


**Figure S2.** Validation of the eight genes on the protein expression level based on HPA database.


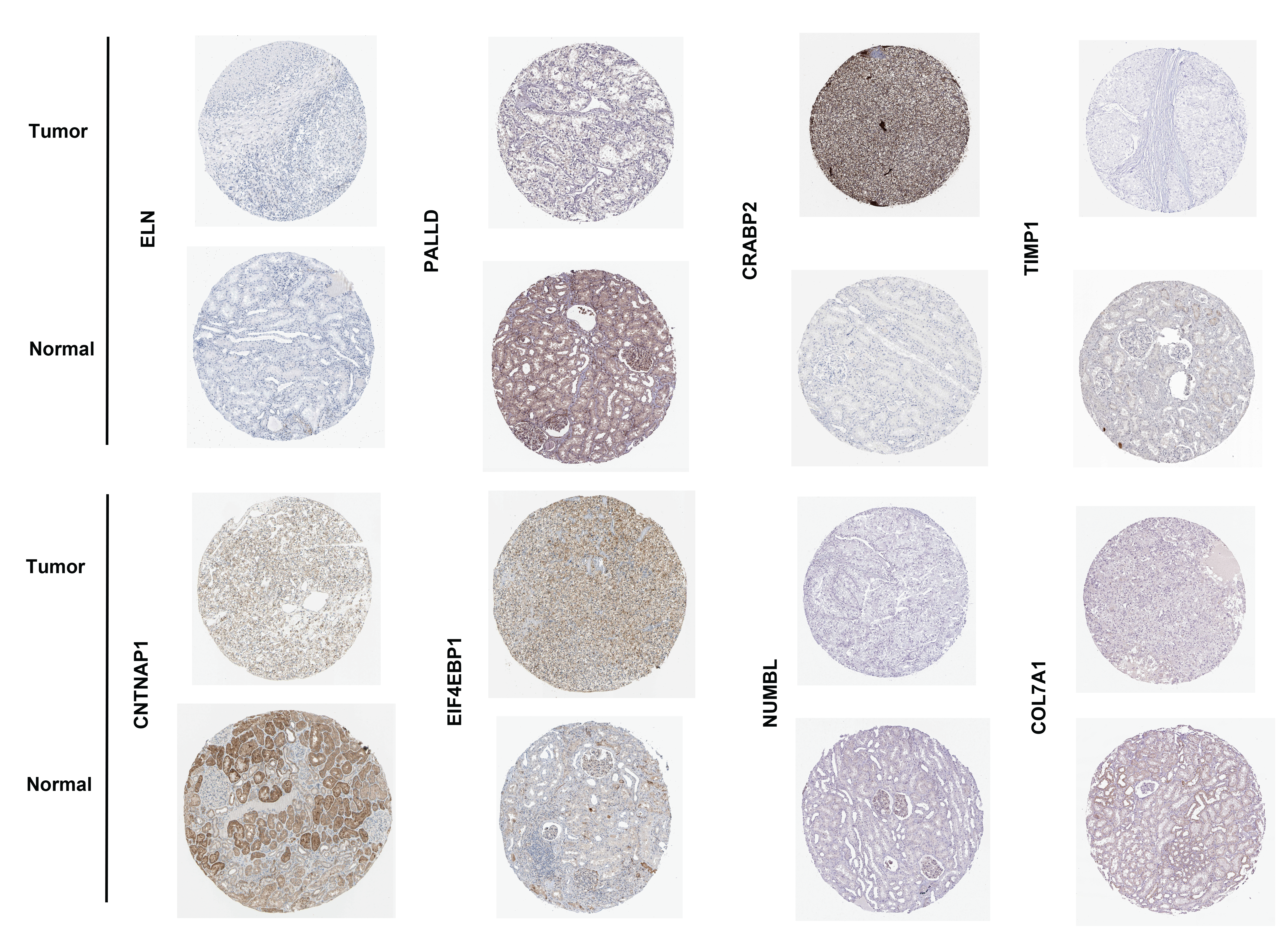

Supplement: Supplementary file 1 — Additional file 1: Figure S1. Estimation of the number of inflammatory response-based unsupervised classes in TCGA-KIRC dataset. (A) Consensus CDF curves show consensus distributions for each k. (B) Delta area plots exhibit the relative alteration in area under CDF curve for k relative to k-1. (C) Item tracking plots display the consensus cluster of items at each k. Figure S2. Validation of the eight genes on the protein expression level based on HPA database. [file 12920_2023_1687_MOESM1_ESM.doc]
